# Clinical Performance of an Enhanced Monofocal IOL Bilaterally Implanted in Patients Targeted for Monovision: A Prospective Study

**DOI:** 10.3390/jcm15020875

**Published:** 2026-01-21

**Authors:** Javier García-Bella, Celia Villanueva, Nuria Garzón, Bárbara Burgos-Blasco, Beatriz Vidal-Villegas, Julián García-Feijoo

**Affiliations:** 1 Department of Ophthalmology, Clinico San Carlos Hospital, 28040 Madrid, Spain; javier.garciabll@gmail.com (J.G.-B.); cevill02@ucm.es (C.V.); bburgos171@hotmail.com (B.B.-B.); beatrizvidalvillegas@gmail.com (B.V.-V.); jgarciafeijoo@hotmail.com (J.G.-F.); 2 Faculty of Optics and Optometry, Complutense University of Madrid, 28037 Madrid, Spain

**Keywords:** monovision, presbyopia, intraocular lens, spherical aberration

## Abstract

**Background/Objectives**: The purpose of the study is to assess visual and refractive outcomes and patient satisfaction after bilateral implantation of an enhanced monofocal intraocular lens (IOL) in a monovision configuration. **Methods**: Prospective, monocentric, non-comparative study including adults 21 years or older, with astigmatism less than 1.50 D, who were suitable for bilateral cataract surgery targeted with −1.00 D monovision. Participants were implanted with the RayOne EMV and followed up for three months. Outcome measures included refraction, monocular and binocular uncorrected distance visual acuity (UDVA), corrected distance visual acuity (CDVA), uncorrected and distance-corrected intermediate visual acuity (UIVA and DCIVA) at 66 cm and 80 cm, binocular defocus curve, and CatQuest-9SF questionnaire. **Results**: Sixty eyes of thirty patients were included. Postoperative spherical equivalent (SEQ) was −0.16 ± 0.29 D in the dominant eyes and −1.24 ± 0.43 D in the non-dominant eyes. Binocularly, mean UDVA at 4 m was −0.01 ± 0.07 and 0.1 logMAR or better in all patients. Mean binocular UIVA at 66 cm was 0.08 ± 0.08 and 0.2 logMAR or better in 92.9% of patients. Binocular UDVA was statistically significantly improved compared to monocular UDVA of the dominant eye targeted for distance (*p* < 0.001). Similarly, binocular UIVA was statistically significantly improved compared to monocular UIVA of the non-dominant eye targeted for −1.00 D (*p* < 0.001). A total of 96.6% of patients were satisfied with their sight. **Conclusions**: Bilateral implantation of an enhanced monofocal IOL in a monovision configuration provided excellent binocular uncorrected vision at distance and intermediate ranges, demonstrating effective binocular summation and a high level of patient satisfaction.

## 1. Introduction

The advancements in surgical techniques and intraocular lens (IOL) technology, combined with a growing emphasis on meeting patients’ visual expectations, have shifted the goal of cataract surgery beyond merely restoring distance vision. Increasingly, the objective is to reduce dependence on glasses by addressing a broader range of visual needs. In today’s digital age, patients often seek functional vision not only for far distances but also for intermediate tasks such as working on computers or using smartphones. A well-established strategy to achieve this is the monovision approach with monofocal IOLs, in which the dominant eye is corrected for distance (emmetropia) and the non-dominant eye is adjusted to a mild degree of myopia [1,2,3,4]. This approach has been shown to provide an improvement in intermediate vision [5] and a high patient satisfaction rate [6]. This approach offers a cost-effective and low-risk solution for improving both distance and intermediate vision, while minimizing the risk of dysphotopsias. Nevertheless, using standard monofocal IOLs in a monovision configuration presents certain drawbacks, such as reduced patient tolerance due to anisometropia, diminished binocular depth perception, and symptoms of visual fatigue or asthenopia. 

Enhanced monofocal intraocular lenses (IOLs) are specifically developed to broaden the visual range, offering improved unaided intermediate vision while preserving distance acuity similar to that provided by conventional monofocal lenses. Research to date indicates that implanting these lenses bilaterally can be an effective strategy when used within a monovision framework [7,8,9], providing additional benefits over standard monofocal IOLs, particularly at near and intermediate distances [8]. Zheleznyak et al. [10] used a binocular adaptive optics simulator to compare two monovision strategies, both with an anisometropic offset of −1.5 D: traditional monovision and modified monovision, in which spherical aberration was additionally induced in the non-dominant eye. They reported that modified monovision improved through-focus visual acuity and binocular depth-of-focus compared with traditional monovision. Vandermer et al. [11] also concluded that modified monovision, which includes the induction of spherical aberration, was more effective than traditional monovision in increasing binocular depth-of-focus and improving intermediate vision. 

The RayOne EMV RAO200E (Rayner, Worthing, UK) is an enhanced monofocal intraocular lens designed to extend the visual range through the induction of controlled positive spherical aberration. This lens can be used with or without a monovision approach, allowing customization of the patient’s visual range. Optical bench analyses have shown that the RayOne EMV delivers a broader range of vision compared to traditional monofocal IOLs [12,13]. Early pilot work by Madhivanan et al. [14] provided the first evidence of good distance and intermediate acuity, with normal contrast sensitivity and no device-related complications at short-term follow-up. Yet, their cohort consisted predominantly of bilaterally emmetropic eyes, leaving open the question of how the lens behaves under intentional anisometropia.

Łabuz et al. [15] assessed its optical performance in a monovision configuration and confirmed the extension of the binocular visual range with optical bench testing. However, binocular summation needs to be assessed on patients implanted with the RayOne EMV. The clinical safety and performance of the EMV IOL, whether targeted for bilateral emmetropia [16] or used in a mini-monovision approach, have also been confirmed in previous studies [17,18,19]. However, the achievable additional gain of clear visual range with higher levels of monovision remains uncertain. 

In this clinical study, patients were implanted bilaterally with the RayOne EMV in a monovision configuration, with the dominant eye targeted for emmetropia and the non-dominant eye targeted with −1.00 D of myopia, in order to assess the range of vision and binocular summation in a monovision configuration. Visual and refractive outcomes, as well as patient satisfaction, were also evaluated. 

## 2. Materials and Methods

This was a prospective, observational, non-comparative study performed at San Carlos Hospital, in Madrid, Spain. The study was reviewed and approved by the hospital’s Ethics Committee (Approval 21/664-O_P). All patients included in the study signed a written informed consent preoperatively, after being fully informed about the purpose of the study. The study followed the tenets of the Declaration of Helsinki. 

Participants in the study were men and women aged 21 years or older with bilateral cataracts, deemed suitable candidates for bilateral implantation of the RayOne EMV intraocular lens using a monovision approach. Inclusion criteria included preoperative corneal astigmatism of less than 1.50 diopters, a predicted postoperative best-corrected distance visual acuity (CDVA) of 0.18 logMAR or better, demonstrated tolerance to monovision, and an IOL power calculation between +10.0 D and +30.0 D. Patients were excluded if they had any ocular or systemic condition that might compromise visual outcomes. Eyes with a history of corneal refractive surgery were also excluded from the study.

### 2.1. Visits

Patients attended a preoperative visit, one surgical visit for each eye, with a maximal interval of 14 days between surgeries, and three postoperative visits: a telephone call 1 to 2 days after surgery (Day-1), 30 to 60 days after surgery (Month 1), and 100 to 120 days after surgery (Month 3).

### 2.2. Intraocular Lens

The RayOne EMV RAO200E is a non-diffractive, enhanced monofocal aspheric intraocular lens (IOL) designed to improve visual range through the induction of controlled positive spherical aberration—up to 0.15 µm over its 6 mm optic zone—effectively extending vision from distance to intermediate. Its optical architecture also incorporates a blended edge profile that helps minimize longitudinal spherical aberration, preserving contrast sensitivity and visual acuity under mesopic lighting conditions. The lens is composed of Rayacryl, a hydrophilic acrylic material with a refractive index of 1.46 and an Abbe number of 56. It measures 12.5 mm in total diameter and features closed C-loop haptics for stable capsular bag fixation. Additionally, the design includes Amon-Apple enhanced 360° squared edges to reduce the risk of posterior capsular opacification [20]. 

Optical bench evaluations of the RayOne EMV design have shown that, in addition to inducing positive spherical aberration, the lens presents a slightly lower effective power in the central optic zone compared to its nominal power, with a progressive increase in power toward the periphery. This radial redistribution of optical power results in an extended monocular depth-of-focus, as acceptable retinal image quality is maintained across a continuous range of defocus rather than concentrated at a single focal plane [21].

The IOL is fully preloaded in the RayOne injector in the full power range (+10.0 D to +30.0 D in 0.5 D increments) and allows implantation through a 2.2 mm incision. 

### 2.3. Surgical Procedure

All surgeries were carried out using the surgeon’s preferred micro-incision technique under topical anesthesia. Standard phacoemulsification was performed through a 2.2 mm clear corneal incision. 

Preoperative biometry was obtained with the IOLMaster 700 (Carl Zeiss Meditec AG, Jena, Germany), and intraocular lens power calculations were performed using the Barrett Universal II formula. The target refraction was emmetropia in the dominant eye and −1.00 D in the non-dominant eye for a monovision set-up. 

Postoperative medication was prescribed according to hospital protocol, including topical antibiotics and steroids. 

### 2.4. Preoperative and Postoperative Assessments

Eye dominance and tolerance to monovision were assessed preoperatively using a +1.00 D plus lens simulation test. Briefly, while patients fixated on a high-contrast distance optotype, a +1.00 D lens was alternately placed in front of each eye to simulate anisometropia, and patients were asked to report which configuration was more comfortable. The same procedure was repeated for near viewing. The eye that provided greater comfort for distance viewing without the plus lens was designated as the dominant eye and targeted for emmetropia. Patients who reported visual discomfort or intolerance during simulation were not considered suitable candidates for monovision and were excluded from enrollment. Formal phoria or tropia measurements were not part of the study protocol; however, patients with known binocular vision disorders were excluded.

Visual acuity assessments were conducted under photopic lighting conditions (85 cd/m^2^) using ETDRS charts at 100% contrast, with results expressed in logMAR units. Subjective refraction and visual acuity measurements (uncorrected distance visual acuity, UDVA, and corrected distance visual acuity, CDVA) were performed using ETDRS charts calibrated for a four meter testing distance, and no conversion factor to a six meter equivalent was applied. Uncorrected intermediate visual acuity (UIVA) and distance-corrected intermediate visual acuity (DCIVA) were assessed at 66 cm and 80 cm, under both monocular and binocular conditions. DCIVA measurements were obtained using the manifest refraction for distance correction, following standardized protocols recommended in the published literature [22].

Pupil size was measured using the KR-1W wavefront analyser (Topcon, Tokyo, Japan).

Defocus curve was performed with distance correction, binocularly, under photopic conditions (85 cd/m^2^), with defocus values between +2.00 D to −4.00 D in 0.50 D steps. 

Patients completed the CatQuest-9SF questionnaire preoperatively and at the three-month postoperative visit. 

Adverse events were recorded at all visits.

### 2.5. Statistical Analysis

The sample size calculation was based on the assumption of superiority of binocular uncorrected intermediate visual acuity (UIVA) over binocular distance-corrected intermediate visual acuity (DCIVA). Using a two-tailed test with a significance level of 0.05 and a statistical power of 95%, it was determined that a minimum of 25 participants would be required to detect a clinically meaningful difference of at least 0.05 logMAR units. Accounting for an anticipated dropout rate of 15%, the final enrollment target was set at 30 subjects.

Data analysis was conducted using Microsoft Excel for Windows, version 2404. Descriptive statistics, including mean and standard deviation (SD), were used to summarize the outcomes for each variable. A *p*-value of less than 0.05 was considered indicative of statistical significance. Rasch analysis was applied to evaluate the psychometric outcomes of the CatQuest-9SF questionnaire [23]. Lower values are associated with lower levels of difficulty in vision tasks and higher levels of satisfaction.

## 3. Results

A total of 30 patients (60 eyes) were included and bilaterally implanted with the EMV IOL. All patients completed the three-month follow-up. Mean preoperative characteristics are described in Table 1. 

### 3.1. Refractive Results

**Figure 1:**
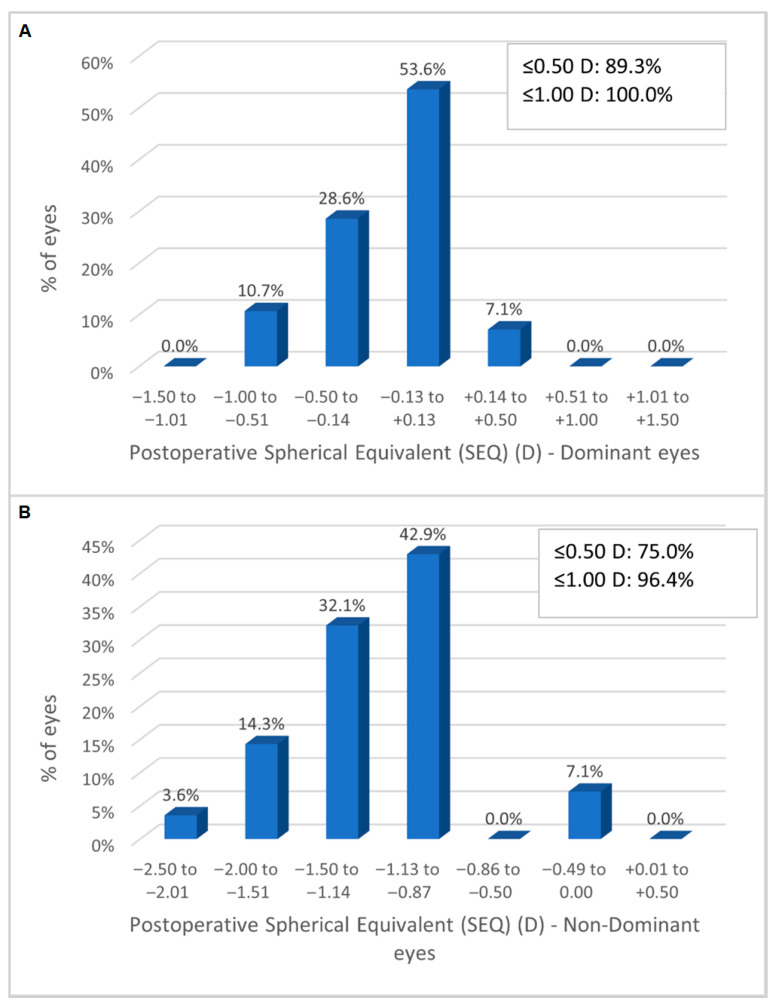
Refractive accuracy: distribution of the SEQ at three months for the dominant eyes (**A**) and non-dominant eyes (**B**).

At three months, the mean postoperative spherical equivalent (SEQ) was −0.16 ± 0.29 D (−1.00 D to +0.25 D) in the dominant eyes and −1.24 ± 0.43 D (−2.38 D to −0.25 D) in the non-dominant eyes. Figure 1A shows the distribution of the SEQ at three months: in the dominant eyes, all eyes were within ±1.00 D of emmetropia, and in the non-dominant eyes, 96.4% of eyes were within ±1.00 D of the −1.00 D myopic target (Figure 1B).

### 3.2. Visual Acuity Results

Mean visual acuity values at one month and three months are shown in Table 2.

At three months, all eyes had a monocular CDVA of 0.04 logMAR or better. 

**Figure 2:**
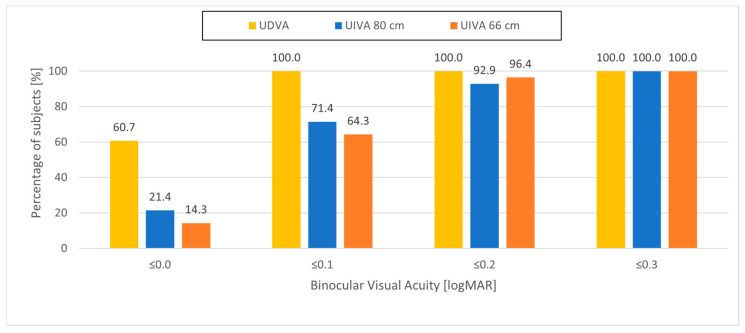
Cumulative distribution of binocular uncorrected visual acuity at distance, intermediate 80 cm, and intermediate 66 cm.

In the dominant eyes targeted for distance, mean monocular UDVA was 0.02 ± 0.09 logMAR. Binocularly, mean UDVA was −0.01 ± 0.07 logMAR and was statistically significantly better than monocular UDVA of the dominant eyes (*p* = 0.004). Binocular UDVA was 0.0 logMAR or better in 60.7% of patients and 0.1 logMAR or better in all patients (Figure 2). 

In the non-dominant eyes, mean monocular UIVA was 0.11 ± 0.11 logMAR at 80 cm and 0.11 ± 0.09 logMAR at 66 cm. Mean binocular UIVA was 0.07 ± 0.09 logMAR at 80 cm and 0.08 ± 0.08 logMAR at 66 cm. There was no statistically significant difference in binocular UIVA between 66 cm and 80 cm (*p* = 0.294). Binocular UIVA was statistically significantly better than monocular UIVA of the non-dominant eyes at 66 cm (*p* = 0.004) and at 80 cm (*p* = 0.003). Binocular UIVA at 80 cm was 0.1 logMAR or better in 71.4% of patients and 0.3 logMAR or better in all patients; binocular UIVA at 66 cm was 0.1 logMAR or better in 64.3% of patients and 0.3 logMAR or better in all patients (Figure 2). 

Mean binocular DCIVA was 0.20 ± 0.06 logMAR at 80 cm and 0.27 ± 0.10 logMAR at 66 cm. Binocular UIVA at 80 cm was statistically significant better (*p* < 0.001) than binocular DCIVA at 80 cm by almost one and a half lines; binocular UIVA at 66 cm was statistically significant better (*p* < 0.001) than binocular DCIVA at 66 cm by almost two lines.

### 3.3. Defocus Curve

**Figure 3:**
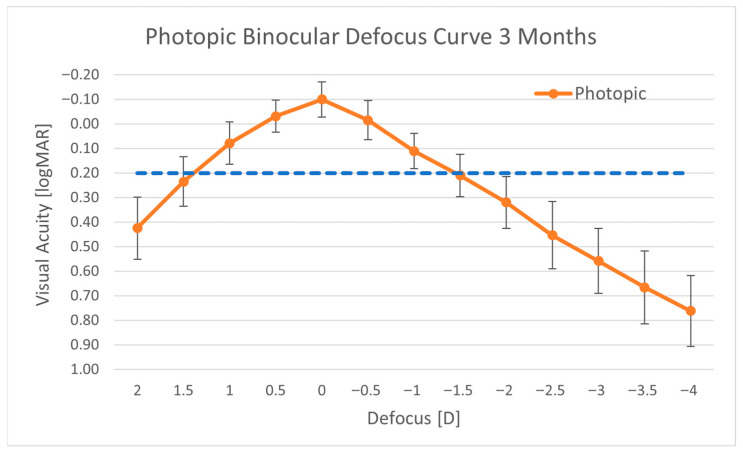
Distance-corrected binocular defocus curve.

The distance-corrected binocular defocus curves (Figure 3) showed a peak at a defocus of 0.00 D (equivalent to 4 m) and a visual acuity of 0.20 logMAR or better over a range of 2.75 D from +1.25 D to −1.50 D. Mean visual acuity at a defocus of −1.50 D, corresponding to DCIVA measured at 66 cm, was 0.21 ± 0.09 logMAR. 

### 3.4. Questionnaires

**Figure 4:**
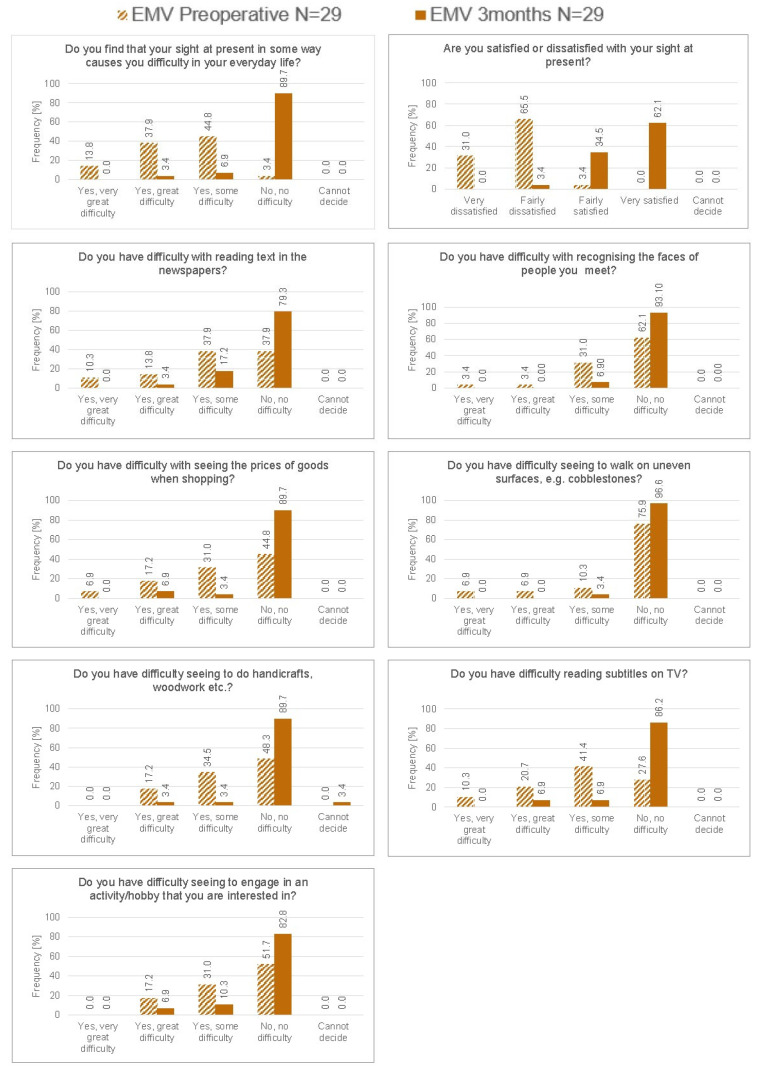
CatQuest-9SF questionnaire.

Outcomes of the CatQuest-9SF questionnaire obtained both preoperatively and at three months are shown in Figure 4. For all tasks, there was a large improvement postoperatively compared to preoperatively. Overall, 90% of patients reported no vision-related difficulties in their everyday life. This was confirmed by the overall Rasch score improving from −1.33 ± 1.33 (range: −3.38 to 1.61) preoperatively to −3.50 ± 0.94 (range: −3.98 to 0.06) at three months postoperatively. The improvements at three months after surgery compared with before surgery, for the overall score and for the score of each individual question, were statistically significant (*p* < 0.05). 

### 3.5. Adverse Events

There were no adverse events. No patients expressed any intolerance to anisometropia. Furthermore, none of the patients reported spontaneously experiencing halos or night vision disturbances during the postoperative visits. 

## 4. Discussion

In this study, we evaluated visual and refractive outcomes, as well as quality of life, in a group of patients who underwent bilateral cataract surgery with implantation of the RayOne EMV IOL in a monovision configuration, with the dominant eye targeted for emmetropia and the non-dominant eye targeted for myopia of −1.00 D. 

Mean binocular UDVA was −0.01 ± 0.07 logMAR and mean binocular UIVA was 0.08 ± 0.08 logMAR at 66 cm and 0.07 ± 0.09 at 80 cm, with all patients seeing 0.1 logMAR or better at distance and 92.9% of patients seeing 0.2 logMAR or better at 66 cm. The fact that binocular UIVA at 66 cm was similar to binocular UIVA measured at 80 cm demonstrates that the range of clear vision was increased towards near vision to at least 66 cm.

The uncorrected distance visual outcomes were comparable to those reported in a previously published historical cohort implanted with the same IOL by the same surgical team, although that cohort was not part of the current study design and had been targeted for emmetropia [16]. In our previous paper [16], binocular UDVA was 0.1 logMAR or better in 95% of patients, compared to 100% in this paper. This suggests that uncorrected distance vision is at least as good in the monovision configuration compared to the emmetropia configuration and, therefore, that the quality of distance vision is not compromised by the monovision set-up. This confirms the results of a previous optical bench study by our group, where we hypothesized that good binocular distance outcomes could be obtained with the RayOne EMV in a monovision approach [23].

The monovision configuration appears to be beneficial in improving vision at 66 cm. Mean binocular UIVA was better in the monovision configuration (0.08 ± 0.08 logMAR) compared to the emmetropia configuration (0.14 ± 0.11), with 64.3% of patients able to see 0.1 logMAR or better with monovision versus 57.0% in the emmetropia configuration. In addition, binocular UIVA was statistically significantly better than DCIVA at both 66 cm and 80 cm, demonstrating that the monovision configuration was effective in improving unaided visual acuity at intermediate distances. These findings are comparable to other studies comparing monovision and emmetropia settings with different enhanced monofocal IOLs. Sandoval et al. [7] reported no statistically significant difference in mean UDVA between the monovision group, with a mean myopia of −0.76 ± 0.10 D, and the emmetropia groups with patients bilaterally implanted with the Tecnis Eyhance; however, UIVA was statistically significantly better in the monovision group. Park et al. [9] also reported outcomes on the Tecnis Eyhance and concluded that binocular uncorrected near visual acuity and spectacle independence rates were significantly better in the mini-monovision group compared to the emmetropia group. In their study, the mean spherical equivalent in the non-dominant eyes was –0.95  ±  0.19 D. 

Another important finding of our work is that we were able to demonstrate a statistically significant improvement in binocular visual acuity compared to monocular visual acuity of each eye. Both binocular UDVA and binocular UIVA were improved compared to monocular UDVA of the dominant eyes and monocular UIVA of the non-dominant eyes, demonstrating effective binocular summation. It is believed that this is due to the monovision settings combined with the increased range of vision provided by the optical design of the RayOne EMV. With the dominant eye targeted for near emmetropia, the RayOne EMV provided functional distance and intermediate vision, whereas a planned mild myopia in the non-dominant eye (mean spherical equivalent −1.24 D) extended the functional range toward near, creating a blended binocular range of vision. The blended binocular fusion was not accompanied by any unwanted symptoms such as asthenopia, intolerance to ametropia, or dysphotopsia. 

An exploratory observation of patients who achieved excellent binocular visual acuity at both distance and intermediate did not reveal a clear relationship with postoperative refractive error or pupil size. Regarding the pupil, no consistent trend was observed in photopic pupil diameter within this subgroup, which aligns with previous optical bench studies suggesting that the depth-of-focus extension induced by positive spherical aberration in enhanced monofocal IOLs remains relatively stable across typical photopic pupil sizes. 

Effective binocular summation under anisometropic conditions may have resulted from the optical properties of the RayOne EMV extending image capabilities in both myopic and hyperopic directions. Defocus curve measurements showed a large extended binocular range of vision, exceeding 1.0 D for a visual acuity of 0.0 logMAR or better and 2.75 D for a visual acuity of 0.20 logMAR or better. There was a good agreement within half a line of visual acuity between the visual acuity measured at −1.50 D defocus (0.21 ± 0.09 logMAR) and the binocular DCIVA measured at the corresponding 66 cm distance (0.27 ± 0.10 logMAR). 

The RayOne EMV demonstrated excellent refractive accuracy in both the dominant eyes targeted for emmetropia (100% of eyes within ±1.0 D) and the non-dominant eyes targeted for myopia (96.4% of eyes within ±1.0 D). However, both groups exhibited a mild systematic myopic shift relative to the intended targets. This was most likely related to the use of a non-optimized A-constant for this specific IOL model in our biometry system at the time of patient enrollment, as postoperative refractive optimization had not yet been implemented. Importantly, this mild myopic bias did not compromise binocular distance visual acuity, likely due to the extended depth-of-focus characteristics of the lens. In contrast, in the non-dominant eyes, the slightly more myopic outcome (mean −1.24 D) may have contributed to the favorable intermediate visual acuity observed at 66 cm, suggesting that small deviations toward greater myopia may further enhance functional intermediate performance within a mini-monovision strategy. These outcomes were consistent with the findings presented in our previous paper, where all eyes were targeted for emmetropia and were within ±1.00 D of target [16]. Both dominant and non-dominant eyes tended to be slightly more myopic than intended. However, good uncorrected distance visual acuity was maintained despite the small myopic refractive error in the dominant eyes (−0.16 ± 0.29 D on average). Additionally, the planned difference of 1.00 D between the two eyes was maintained (1.08 ± 0.43 D), allowing good binocular vision. 

The RayOne EMV lens induces controlled positive spherical aberration. This choice is clinically relevant because the typical human cornea already exhibits a degree of inherent positive spherical aberration. By complementing, rather than neutralizing or reversing, this natural aberration, the RayOne EMV achieves an extended depth-of-focus with a relatively modest total amount of induced aberration [24]. This balanced approach helps preserve the MTF, maintaining distance image quality and contrast sensitivity [19].

Importantly, the positive spherical aberration profile results in an asymmetric defocus response, often described as a hyperopic tail. This feature is particularly beneficial when monovision is targeted. In the eye intentionally left mildly myopic, the hyperopic tail partially compensates for the myopic shift, preserving functional distance and intermediate vision despite the induced defocus. Rather than an abrupt loss of MTF, optical performance remains above the functional visual threshold across a broader defocus range.

When combined binocularly, the elongated defocus curves of the two eyes overlap, allowing smooth binocular summation across distance and intermediate vision, while the myopic eye contributes enhanced intermediate/near performance. Thus, the interaction between positive spherical aberration and induced myopia acts synergistically: spherical aberration extends the depth-of-focus and provides tolerance to refractive offset, while monovision shifts the extended focal region to enhance near vision without substantially compromising distance acuity.

Patient satisfaction was high with 89.7% of patients reporting no difficulty at all in everyday life and 96.6% reporting being satisfied with their sight. The monovision configuration allowed patients to perform near tasks with greater ease. The percentage of patients reporting no difficulty reading a newspaper rose from 58.3% in the emmetropia configuration to 79.3% in the monovision configuration. Similarly, patients reporting no difficulty with handicrafts increased from 79.2% for the emmetropia configuration to 89.7% for the monovision configuration. This was also demonstrated by the significant improvement in the CatQuest-9SF Rasch score for each individual question and the overall score. A recent prospective study by Llovet-Rausell et al. [25] confirmed similarly high satisfaction and functional outcomes in patients implanted with the RayOne EMV using a −1.00 D mini-monovision target. However, their analysis focused mainly on PROMs and contrast sensitivity, without quantifying the relationship between the achieved anisometropia and binocular visual quality. The present work expands on those findings by correlating clinical performance with the achieved refractive offset and demonstrating that the functional range and satisfaction remain stable even when the offset deviates slightly from the intended target, reinforcing the robustness of the mini-monovision approach.

Furthermore, our results are consistent with the improvement in quality of vision expected after implantation of an enhanced monofocal IOL [26]. No patient spontaneously reported halos or night vision disturbances during postoperative visits. However, it must be acknowledged that no validated questionnaire specifically designed to assess dysphotopsias or night-driving symptoms was administered. Therefore, mild photic phenomena, particularly those potentially arising from the summation of retinal images from eyes with different refractive targets and induced spherical aberration, may have been underdetected. Consequently, the present findings should not be interpreted as definitive evidence of complete absence of halos or glare, but rather as an absence of clinically significant or patient-reported symptoms under routine clinical questioning. In their optical bench analysis, Łabuz et al. [15] suggested that the monovision configuration with the RayOne EMV inducing positive spherical aberration might result in an increased halo size. This was not confirmed in this study or in previous studies evaluating the RayOne EMV in a monovision configuration [17,18,19]. 

Our study has some limitations, including the inclusion of patients with corneal astigmatism up to 1.5 D, which reflects our current clinical practice. However, no negative impact on visual acuity outcomes was observed in these patients, with postoperative spherical equivalent remaining within ±1.00 D in nearly all eyes. Additionally, our study also lacks a systematic evaluation of halos and glare. In future studies involving monovision, it would be beneficial to incorporate direct, dysphotopsia-focused questionnaires, particularly those addressing symptoms that may result from the neural summation of images originating from eyes with differing refractive targets. Measuring the binocular defocus curve with −1.00 D myopia in the non-dominant eye would have provided valuable insights into the extent of the increased range of vision in this monovision configuration. Furthermore, assessing stereoscopic and mesopic vision measurement before and after surgery might have been beneficial, although preoperative measurements are often affected by cataract severity and asymmetry in opacification between the two eyes. Lastly, near visual acuity at 40 cm was not measured in this study, as the focus was on intermediate visual acuity at 66 cm and 80 cm. The primary objective of the study was to evaluate functional distance and intermediate vision, reflecting common daily visual demands such as computer use and dashboard viewing. The planned refractive target of −1.00 D was not intended to provide full near correction. Nevertheless, given the achieved mean postoperative SEQ of −1.24 D in the non-dominant eye combined with the extended depth-of-focus effect of the IOL, it is biologically plausible that some degree of functional near vision at 40–50 cm was achieved. This is indirectly supported by the substantial postoperative improvement in CatQuest-9SF items related to reading tasks, such as reading newspapers and price labels. Formal near visual acuity testing and mesopic functional assessments should be included in future studies to better characterize near performance and visual quality under low-light conditions.

## 5. Conclusions

Bilateral implantation of the enhanced monofocal RayOne EMV in a monovision configuration provided excellent binocular UDVA and UIVA, with high patient satisfaction. The increased range of vision provided by the design of the RayOne EMV combined with the monovision configuration, resulted in effective binocular summation and improved binocular vision compared to monocular vision. 

Binocular implantation of the RayOne EMV in a monovision configuration offers surgeons an additional option in routine clinical practice, particularly for patients willing to achieve distance vision comparable to a standard monofocal IOL, while benefiting from improved intermediate vision.

## Figures and Tables

**Table 1:** Preoperative patient characteristics.

	Mean ± SD (Range)
Age (years)	68.4 ± 8.8 (42 to 80)
Gender	Female: 67%; Male: 33%
Dominant eye	Right eye: 50%; Left eye: 50%
SEQ (D)	−1.53 ± 2.68 (−8.50 to +3.50)
IOL power (D)	20.03 ± 2.80 (13.00 to 26.50)
Mean corneal astigmatism (D)	0.70 ± 0.36 (0.15 to 1.46)
Axial length (mm)	23.93 ± 1.01 (21.96 to 25.77)
Anterior chamber depth (mm)	3.34 ± 0.29 (2.85 to 3.93)
IOP (mmHg)	15.6 ± 2.5 (10.0 to 20.0)
Pupil size, photopic (mm)	3.4 ± 0.7 (2.0 to 5.0)
Corneal spherical aberration (Z_4_^0^)	0.31 ± 0.08 (−0.01 to 0.40)

SD: standard deviation; D: diopter; mm; millimeter; mmHg: millimeters of mercury.

**Table 2:** Mean logMAR monocular and binocular uncorrected distance visual acuity (UDVA), corrected distance visual acuity (CDVA), uncorrected intermediate visual acuity (UIVA), and distance-corrected intermediate visual acuity (DCIVA) preoperatively, at Month 1 (1 M) and Month 3 (3 M) postoperatively. The table able shows the mean value, standard deviation, and range.

	Preoperatively	Month 1	Month 3	*p*-Value (1 M vs. 3 M)
Monocular CDVA (*n* = 60)	0.23 ± 0.20 (−0.06 to 0.90)	−0.04 ± 0.06 (−0.20 to 0.06)	−0.07 ± 0.06 (−0.20 to 0.04)	<0.001
**Binocular visual acuity outcomes (*n* = 30)**				
CDVA	0.10 ± 0.13 (−0.10 to 0.58)	−0.08 ± 0.06 (−0.20 to 0.02)	−0.10 ± 0.07 (−0.28 to 0.00)	0.0221
UDVA	0.48 ± 0.28 (0.00 to 1.02)	0.01 ± 0.08 (−0.10 to 0.28)	−0.01 ± 0.07 (−0.16 to 0.10)	0.0675
UIVA (80 cm)	0.55 ± 0.22 (0.22 to 0.96)	0.07 ± 0.07 * (−0.04 to 0.20)	0.07 ± 0.09 ** ^∆^ (−0.14 to 0.26)	0.9886
UIVA (66 cm)	0.53 ± 0.23 (0.10 to 0.96)	0.11 ± 0.09 * (−0.08 to 0.26)	0.08 ± 0.08 ** ^∆∆^ (−0.10 to 0.22)	0.9090
DCIVA (80 cm)	0.36 ± 0.10 (0.12 to 0.58)	0.21 ± 0.08 (0.10 to 0.38)	0.20 ± 0.06 ^∆^ (0.10 to 0.32)	0.9090
DCIVA (66 cm)	0.41 ± 0.11 (0.24 to 0.68)	0.27 ± 0.08 (0.16 to 0.44)	0.27 ± 0.10 ^∆∆^ (0.12 to 0.60)	0.5812
**Monocular visual acuity outcomes in dominant eye (*n* = 30)**				
UDVA	0.58 ± 0.29 (0.14 to 1.08)	0.05 ± 0.10 (−0.08 to 0.48)	0.02 ± 0.09 (−0.14 to 0.26)	0.1627
UIVA (80 cm)	0.67 ± 0.29 (0.06 to 1.24)	0.25 ± 0.11 (0.02 to 0.46)	0.24 ± 0.12 (0.04 to 0.56)	0.3160
UIVA (66 cm)	0.68 ± 0.28 (0.20 to 1.14)	0.31 ± 0.11 (0.12 to 0.56)	0.31 ± 0.13 (0.08 to 0.68)	0.4660
**Monocular visual acuity outcomes in non-dominant eye (*n* = 30)**				
UDVA	0.67 ± 0.30 (0.10 to 1.08)	0.30 ± 0.21 (−0.10 to 0.68)	0.29 ± 0.16 (−0.02 to 0.56)	0.2050
UIVA (80 cm)	0.73 ± 0.25 (0.32 to 1.24)	0.13 ± 0.13 (−0.06 to 0.58)	0.11 ± 0.11 (−0.14 to 0.38)	0.1251
UIVA (66 cm)	0.73 ± 0.25 (0.10 to 1.18)	0.12 ± 0.10 (−0.02 to 0.36)	0.11 ± 0.09 (−0.10 to 0.40)	0.3778
DCIVA (80 cm)	0.47 ± 0.23 (0.12 to 1.30)	0.29 ± 0.08 (0.16 to 0.52)	0.26 ± 0.07 (0.14 to 0.38)	0.1658
DCIVA (66 cm)	0.51 ± 0.18 (0.22 to 1.12)	0.35 ± 0.08 (0.22 to 0.60)	0.31 ± 0.07 (0.18 to 0.42)	0.0880

*p*-values: * *p* = 0.002 comparing UIVA 80 cm and UIVA 66 cm at Month 1; ** *p* = 0.294 comparing UIVA 80 cm and UIVA 66 cm at Month 3; ^∆^ *p* < 0.001 comparing DCIVA 80 cm vs. UIVA 80 cm at Month 3; ^∆∆^ *p* < 0.001 comparing DCIVA 66 cm vs. UIVA 66 cm at Month 3.

## Data Availability

The data can be requested by email from the corresponding author.
